# Taurine Alleviates the Progression of Diabetic Nephropathy in Type 2 Diabetic Rat Model

**DOI:** 10.1155/2014/397307

**Published:** 2014-02-23

**Authors:** Jang Hyun Koh, Eun Soo Lee, Miri Hyun, Hong Min Kim, Yoon Jung Choi, Eun Young Lee, Dhananjay Yadav, Choon Hee Chung

**Affiliations:** ^1^Center for Health Promotion, Samsung Medical Center, Sungkyunkwan University School of Medicine, Seoul 135-740, Republic of Korea; ^2^Department of Internal Medicine, Yonsei University Wonju College of Medicine, 162 Ilsan-Dong, Wonju, Gangwon-Do 220-701, Republic of Korea; ^3^Department of Internal Medicine, Soonchunhyang University Cheonan Hospital, Cheonan 330-721, Republic of Korea; ^4^Department of Radiology, Kangbuk Samsung Hospital, Sungkyunkwan University School of Medicine, Seoul 110-746, Republic of Korea

## Abstract

The overexpression of vascular endothelial growth factor (VEGF) is known to be involved in the pathogenesis of diabetic nephropathy. In this study, the protective effects of taurine on diabetic nephropathy along with its underlying mechanism were investigated. Experimental animals were divided into three groups: LETO rats as normal group (*n* = 10), OLETF rats as diabetic control group (*n* = 10), and OLETF rats treated with taurine group (*n* = 10). We treated taurine (200 mg/kg/day) for 20 weeks and treated high glucose (HG, 30 mM) with or without taurine (30 mM) in mouse cultured podocyte. After taurine treatment, blood glucose level was decreased and insulin secretion was increased. Taurine significantly reduced albuminuria and ACR. Also it decreased glomerular volume, GBM thickness and increased open slit pore density through decreased VEGF and increased nephrin mRNA expressions in renal cortex. The antioxidant effects of taurine were confirmed by the reduction of urine MDA in taurine treated diabetic group. Also reactive oxygen species (ROS) levels were decreased in HG condition with taurine treated podocytes compared to without taurine. These results indicate that taurine lowers glucose level via increased insulin secretion and ameliorates the progression of diabetic nephropathy through antifibrotic and antioxidant effects in type 2 diabetes rat model.

## 1. Introduction

Diabetes mellitus is a condition in which lower state of antioxidant has been observed [1]. The most common microvascular complication of diabetes mellitus is diabetic nephropathy [2]. The mechanisms involved in the pathogenesis of diabetic nephropathy are variable and many growth factors initiate diabetic renal complications [3]. Among growth factors, vascular endothelial growth factor (VEGF) plays a part in this pathogenesis [4]. The expression of VEGF increases in extremely vascularized and quickly growing tumors [5]. VEGF is known to increase vascular permeability to macromolecules [6] and its expression is localized to the epithelial glomerular cells, podocytes, distal tubules, and renal collecting duct in the normal kidney [7]. VEGF is especially increased during the early stages of diabetic nephropathy [8]; therefore, reducing VEGF overexpression may ameliorate diabetic renal disease [9].

Proteinuria is one of the diagnostic criteria for diabetic nephropathy. Recent studies have focused on changes to the glomerular basement membrane (GBM) structure [10, 11]. Nephrin is a podocyte-specific protein [12] and its expression is reduced in the early stages of proteinuria in diabetic patients [13].

Taurine (2-aminoethane sulphonic acid) is present in most mammalian tissue and have various effects such as osmoregulation, bile acid conjugation, cell proliferation, and the viability and prevention of oxidant induced tissues injury. In biological system, taurine has antioxidant effect to stabilize biomembranes, reduce the malondialdehyde, and scavenge reactive oxygen species [14]. It is another candidate for the treatment of diabetic nephropathy.

Both plasma and platelet taurine concentrations are decreased in type 1 diabetic patients and these levels increase to normal after oral taurine administration [15]. Since Trachtman et al. reported marked reductions in proteinuria in streptozotocin- (STZ)-induced diabetic rats with decreased renal lipid peroxidation after oral supplementation of taurine [16], several reports about the effects of taurine on diabetic nephropathy showed the results of improvements in oxidative stress [17–20] and reductions in TGF-*β* expression [20]. Recently, taurine administration prevented the occurrence and development of diabetic nephropathy by decreasing blood glucose, improving lipid metabolism and glomerular basement membrane metabolism [21]. These effects of taurine on diabetic nephropathy have not been fully demonstrated and are currently under investigation.

In this study we investigated whether taurine could improve diabetic nephropathy or not and then would like to know the mechanism how it can ameliorate diabetic kidney disease.

## 2. Materials and Methods

### 2.1. Reagents

Taurine was purchased from Sigma-Aldrich Chemical Company (St. Louis, MO, USA).

### 2.2. Treatment of Experimental Animals

All experiments were performed under the approval of the Institutional Animal Care and Use Committee (IACUC) of Yonsei University at Wonju Campus. Ten male Long-Evans-Tokushima-Otsuka (LETO, Otsuka Pharmaceutical Co., Ltd., Tokushima, Japan) rats, were used as nondiabetic controls, and 30 male Otsuka-Long-Evans-Tokushima-Fatty (OLETF) rats were used for a type 2 diabetes mellitus model and were purchased from Otsuka Pharmaceutical Co., Ltd. (Tokushima, Japan). At 25 weeks of age the rats were classified into three groups as follows: normal control group, diabetic control group, and taurine-treated diabetic group (200 mg/kg/day). Animals were housed at a constant temperature (20 ± 2°C) and humidity level (50–60%) with a 12 hours light and dark cycle. The animals had free access to water and 20% sucrose containing rat chow (Junsei Chemical Company Ltd., Tokyo, Japan) until 45 weeks of age. The chemical treated groups received their treatments by an oral gavage tube from 26 to 45 weeks of age. At 15, 25, 35, and 45 weeks of age, body weights were measured. At 25, 35, and 45 weeks of age, 24 hours urine was collected with metabolic cages in order to measure the urinary albumin (Exocell Nephrat II; Exocell Inc., Philadelphia, PA, USA) and creatinine (Exocell The Creatinine Companion; Exocell Inc.). We then calculated a urinary albumin creatinine ratio (ACR). At 45 weeks of age, all rats were anesthetized with Zoletil50 (Virbac Laboratories, Carros, France) by intraperitoneal injection. Blood samples were taken by cardiac puncture and collected in test tubes containing heparin solution and centrifuged at 1,500 g for 10 minutes in order to obtain plasma. The plasma was stored at −80°C until use. After perfusion with 0.9% saline, both kidneys were removed. One kidney was stored at −80°C for analysis of mRNA and protein expression and the other kidney was embedded with 4% paraformaldehyde for histological examination.

### 2.3. Measurements of Fasting Blood Glucose, Plasma Insulin, and Adiponectin

Twelve-hour fasting blood glucose levels were analyzed with the LifeScan SureStep (Lifescan, Burnby, Canada) using tail vein blood. Plasma glucose disposal rate (short insulin tolerance test, Kitt; %/min), which indicates the time necessary to reduce the basal glucose level by 50%, was calculated as 0.693/*t*_1/2_, where *t*_1/2_ was determined from the slope of the glycemic concentrations from 3 to 15 minutes after intravenous injection of regular insulin (1 U/kg). Plasma levels of insulin (Shibayagi Co., Shibukawa, Japan) and adiponectin (AdipoGen Inc., Seoul, Korea) were measured using an ELISA kit. Beta-cell function was determined by homeostasis model assessment of beta-cell function (HOMA-*β*) which was calculated as follows [22]: fasting insulin (*μ*U/mL) × 20/[fasting glucose (mM/L) − 3.5].

### 2.4. Determination of Urinary Malondialdehyde (MDA) Levels

A rapid and sensitive fluorometric HPLC method was used to measure urine MDA, which was analyzed by the NeoDin Medical Institute (Seoul, Korea). Urine samples were treated with 0.1125N PCA and 40 mM 2-TBA and subjected to heat at 97°C for 1 hour. To stop the reaction, the samples were put on ice for 20 minutes and then methanol and 20% TCA buffer were added. Samples were mixed and centrifuged at 13,000 g for 6 minutes; then the supernatant was transferred to the insert vial. The fluorescence detector was set to an excitation wavelength of 525 nm and an emission wavelength of 560 nm. The run time was 2 minutes and the flow rate was 1 mL/min.

### 2.5. Histological Examination of Kidney

Paraffin-embedded kidney tissue was cut into sections 4 *μ*m thick and stained with periodic acid-Schiff (PAS). We examined these sections with an optical microscope that was equipped with a charge coupled device camera (Pulnix, Sunnyvale, CA, USA) in order to obtain the pictures of glomeruli from the outer and middle thirds of the renal cortex. We measured glomerular areas using an image analysis system (GmbH, SIS, Minster, Germany) and calculated glomerular volume using the Weibel and Gomez formula as follows [23]: Glomerular volume (Gv) = Area^1.5^ × 1.38/1.01 (1.38: shape coefficient, 1.01: size distribution coefficient). About 30 glomeruli were observed in the kidney sections of each rat.

For the evaluation of the ultrastructure of the glomeruli, kidney tissue was thin sectioned and examined under a JEOL transmission electron microscope (JEM-1200EX II, JEOL Ltd., Tokyo, Japan). Electron micrographs were taken at ×30 K for each sample. The numbers of slit pores were counted and divided by the GBM length (10 *μ*m) to arrive at the linear density using an image analysis system. About 10 glomeruli were observed in the kidney sections of each rat.

### 2.6. Immunohistochemical Staining for VEGF

Four percent paraformaldehyde-fixed kidney tissues were embedded in paraffin. The kidney tissues were prepared into 4 *μ*m sections and placed on a microscope slide. Paraffin was then removed at 60°C for 1 hour, followed by dehydration in xylene. The sections were subjected to graded alcohols, immersed in distilled water (PBS-T) for 5 minutes, and then incubated in 20 *μ*g/mL proteinase K solution (DAKO, Glostrup, Denmark) for 15 minutes. The slides were washed with PBS-T and incubated in Ultra V block (DAKO) for 5 minutes in room temperature.

Polyclonal anti-VEGF (Santa Cruz Biotechnology Inc., Santa Cruz, CA, USA) was added in a 1 : 100 dilution and the sections were kept overnight at 4°C. The slides were washed with PBS-T and biotinylated secondary antibody (Santa Cruz Biotechnology Inc.) was added. The antibody binding was visualized using the avidin and biotinylated horseradish peroxidase reaction. To evaluate the VEGF staining, slides were observed using a light-microscope adhered to a charge-coupled camera device (Pulnix, Sunnyvale, CA, USA). We measured the optical density of stained VEGF using an image analysis system (GmbH, SIS, Minster, Germany). About 20 glomeruli were observed in the kidney sections of each rat.

### 2.7. Expression of Nephrin and VEGF mRNA in the Kidney

Total RNA was isolated and purified from the kidney cortex and concentrations were determined using a microspectrophotometer. For cDNA synthesis, reverse transcription was performed with 1 *μ*g of RNA. The cDNA was prepared using these samples as templates according to protocols provided with a commercially available QuantiTect Reverse Transcription kit (QuantiTect reverse transcription kit; Qiagen, Hilden, Germany). For quantitative real-time PCR, SYBR Green PCR master mix (Applied Biosystem, Foster City, CA, USA) was used in an ABI PRISM 7900HT Sequence Detection System (Applied Biosystems). Quantitative real-time PCR was activated and cDNA was denatured by a preincubation for 15 minutes at 95°C; the template was then amplified for 35 cycles with denaturation for 15 seconds at 94°C, annealing of primers at 58°C for 30 seconds, and extension at 72°C for 30 seconds.

### 2.8. Immunoblot Analysis of the Kidney

To analyze the protein expression of VEGF in the kidney tissues, a western blot analysis was performed. The kidney cortex samples were homogenized by the TissueLyser II (QIAGEN GmbHm Haan, Germany) in a RIPA lysis buffer (Thermo Scientific, Rockford, IL, USA). The protein samples were heated for 5 minutes at 95°C and then electrophoresed on SDS-PAGE (10%) gels and transferred to polyvinylidene difluoride (PVDF) membrane (Immobilon; Millipore, Bedford, MA, USA) for 2 hours at 250 mA. The membrane was blocked by 5% skim milk for 1 hour at room temperature and then incubated with rabbit anti-*β*-actin (1 : 1000) anti-VEGF (1 : 500) and antinephrin antibodies (1 : 1000) (Santa Cruz Biotechnology Inc., Santa Cruz, CA, USA) overnight at 4°C. The membrane was washed 5 times in Tris-buffer saline/0.1% Tween 20 prior to 1 hour of probing with horseradish peroxidase-conjugated secondary antibody. The blots were detected using an enhanced chemiluminescent substrate (Thermo Fisher Scientific Inc., Fremont, CA, USA).

### 2.9. Cell Cultures

Conditionally immortalized mouse podocytes (provided kindly by Dr. Mundel of the University of Harvard, Cambridge, MA, USA) were cultured at 37.5°C without *γ*-interferon in DMEM containing 5.5 mM glucose. Differentiated podocytes were synchronized into quiescence by growing cells in serum-free medium for 24 hours. The cultured podocytes were treated with high glucose media (30 mM), high glucose media with taurine (30 mM). After 24 hours, we measured nephrin and VEGF mRNA expression.

### 2.10. Reactive Oxygen Species (ROS) Production in Podocytes

For the measurement of ROS production, cultured podocytes were added to dichlorofluorescein diacetate (DCF-DA) at a concentration of 10 *μ*M at 37.5°C for 30 minutes and then analyzed for fluorescence using a fluorometer (GloMax-Multi Jr Single Tube Multimode Reader, Promega Corporation, Madison, WI, USA).

## 3. Data Analysis

Data are expressed as means ± SEM. Statistical analyses were conducted with SPSS version 18.0 for windows using a one-way ANOVA and Tukey's test. *t*-tests were used for experiments with only two groups. Differences were considered significant at *P* < 0.05.

## 4. Results

### 4.1. Clinical and Biochemical Characteristics of Experimental Rats

**Figure 1:**
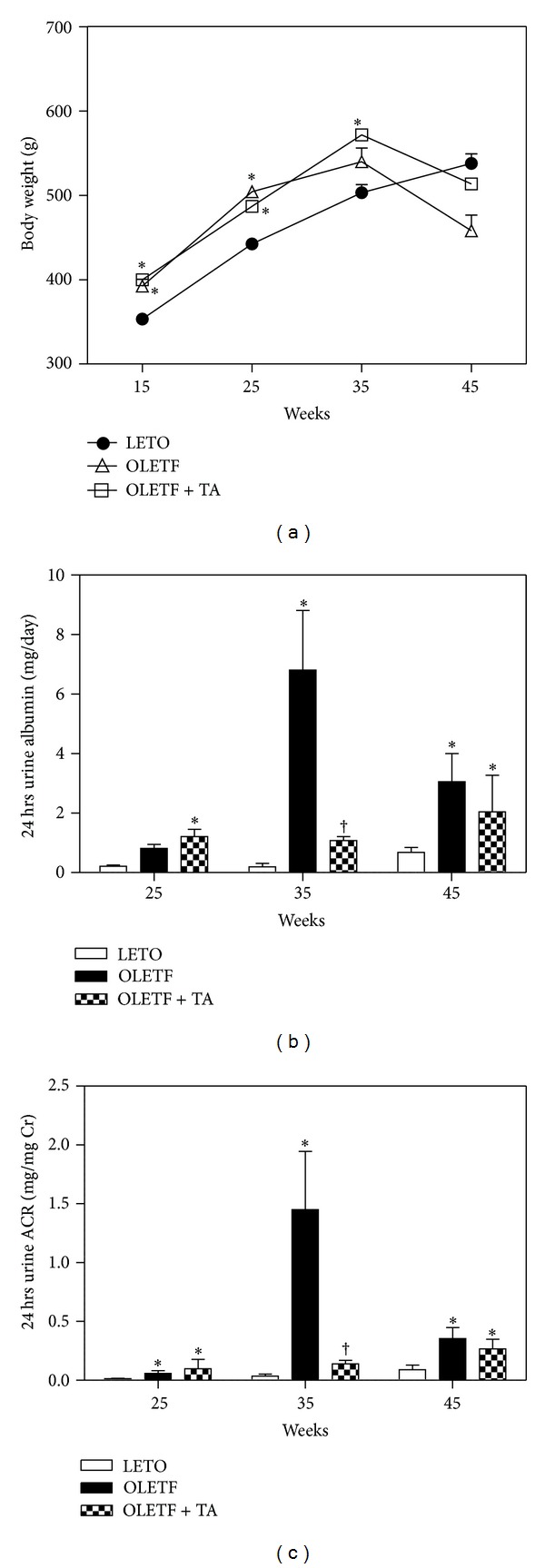
Changes of body weights, 24 hours urine albumin, and ACR in experimental rats on the basis of the duration of diabetes mellitus. (a) The body weights of OLETF and taurine-treated groups were decreased compared to LETO after 35 weeks of age. (b) Urine albumin levels were significantly lower at 35 weeks of age in the taurine-treated diabetic group compared to the diabetic control group. (c) 24 hours urine ACR significantly decreased in the taurine-treated diabetic group compared to the diabetic control group. ACR, albumin creatinine ratio; LETO, normal control group; OLETF, diabetic control group; OLETF + TA, taurine-treated diabetic group. Data are expressed as mean ± SEM. *P < 0.05 compared with LETO; ^†^ *P* < 0.05 compared with OLETF.

Fasting blood glucose levels significantly increased in the diabetic control group compared to the normal control group and significantly decreased in the taurine-treated diabetic control group. Insulin levels significantly decreased in the diabetic OLETF and diabetic with taurine groups compared to the normal control group. Among the diabetic groups, insulin levels were significantly higher in the taurine-treated diabetic group (OLETF + TA) compared to the diabetic control group (OLETF). HOMA-*β* was increased in the taurine-treated diabetic group compared to the diabetic control group. Adiponectin significantly decreased in the diabetic control group compared to the normal control group and increased in the taurine-treated OLETF group, although this result was not statistically significant (Table 1). While the body weights of the animals in the normal control group (LETO) increased continuously until 45 weeks of age, those of the animals in the diabetic OLETF and diabetic with taurine groups decreased slightly after 35 weeks of age (Figure 1(a)). 24 hours urine albumin and ACR significantly decreased in the taurine-treated diabetic group compared to the diabetic control group at 35 weeks; however these differences were not statistically different at 45 weeks (Figures 1(b) and 1(c)).

### 4.2. Histological Changes of Renal Glomeruli

**Figure 2:**
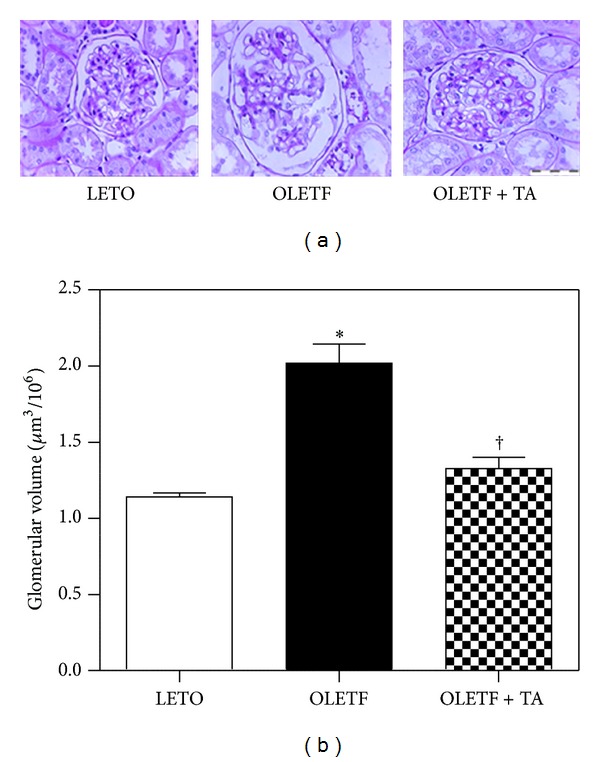
Morphological characteristics of renal glomeruli in the three groups. (a) Cross-sectioned glomeruli were stained with periodic acid-Schiff (×400). (b) The volume of renal glomeruli in the taurine-treated diabetic group decreased compared the diabetic control group. LETO, normal control group; OLETF, diabetic control group; OLETF + TA, taurine-treated diabetic group. Data are expressed as mean ± SEM. *P < 0.05 compared with LETO; ^†^ *P* < 0.05 compared with OLETF.

The weight of the right kidney increased in the diabetic groups and significantly decreased in the taurine-treated diabetic group (0.42 ± 0.02 g) than in the diabetic control group (0.51 ± 0.02 g). However, there was no statistical significance in the difference in left kidney weight between the diabetic control (0.52 ± 0.02 g) and taurine-treated diabetic groups (0.43 ± 0.03 g). The calculated glomerular volume of the diabetic control group significantly increased compared to the normal control group. In the taurine-treated diabetic group the volume of the renal glomerulus was significantly diminished compared to the diabetic control group (Figure 2).

### 4.3. Electron Microscopic Morphometry of the Open Slit Pores Number and Glomerular Basement Membrane Thickness

**Figure 3:**
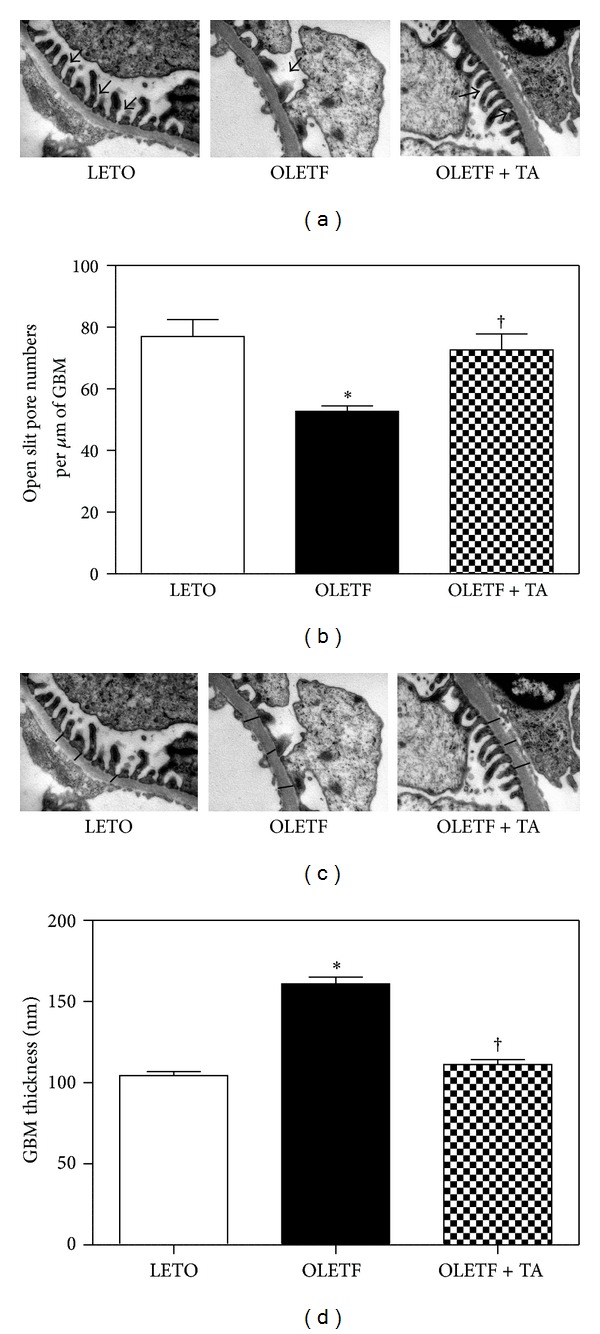
Changes in the numbers of open slit pores and GBM thickness among the three groups. (a) Electron microscopy demonstrated open slit pores (arrows) between the foot processes of the podocytes (×30 K). (b) The numbers of open slit pores significantly increased following taurine treatment when compared to the diabetic control group. (c) Electron microscopy revealed the GBM thickness. Arrows indicate the thickness of the GBM (×30 K). (d) The taurine-treated diabetic group had significantly decreased GBM thickness compared to the diabetic control group. GBM, glomerular basement membrane; LETO, normal control group; OLETF, diabetic control group; OLETF + TA, taurine-treated diabetic group. Data are expressed as mean ± SEM. *P < 0.05 compared with LETO; ^†^ *P* < 0.05 compared with OLETF.

The numbers of open slit pores decreased in the diabetic control group compared to the normal control group and significantly increased in the taurine-treated diabetic group compared to the diabetic control group (Figures 3(a) and 3(b)). The thickness of the GBM was increased in the diabetic control group compared to the normal control group and significantly decreased in the taurine-treated diabetic group, approaching that of the normal control group (Figures 3(c) and 3(d)).

### 4.4. The mRNA Expression of VEGF, Nephrin, and Type IV Collagen in the Renal Cortex

**Figure 4:**
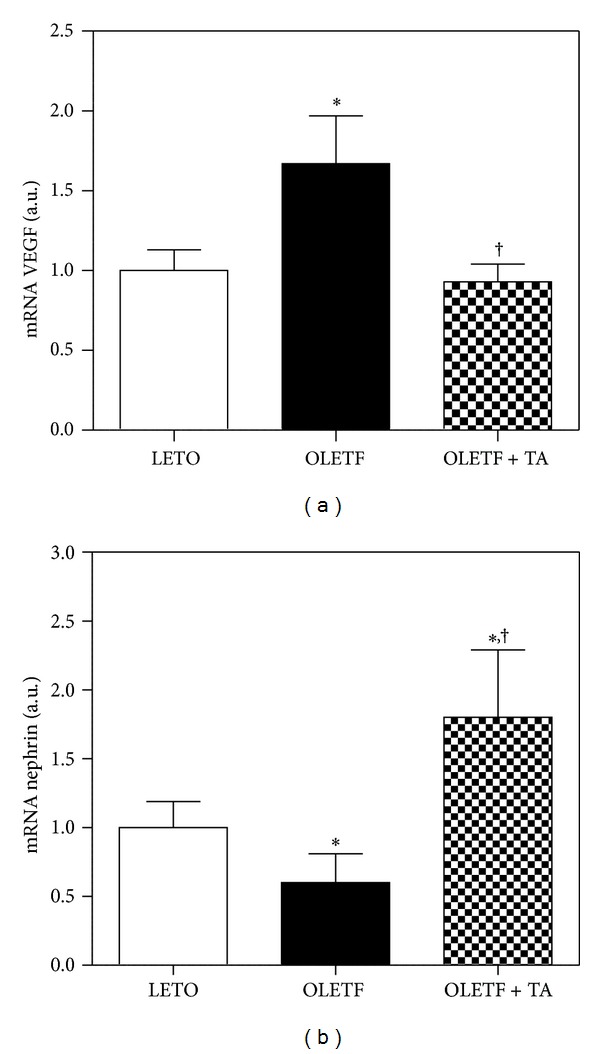
Differences in VEGF and nephrin mRNA expression in the kidney. (a) Renal VEGF mRNA expression decreased in the taurine-treated diabetic group compared to the diabetic control group. (b) Renal nephrin mRNA expression increased in response to taurine treatment. LETO, normal control group; OLETF, diabetic control group; OLETF + TA, taurine-treated diabetic group. Data are expressed as mean ± SEM. *P < 0.05 compared with LETO; ^†^ *P* < 0.05 compared with OLETF.

The renal VEGF mRNA expression, according to quantitative RT-PCR, decreased significantly in the taurine-treated diabetic group compared to the diabetic control group (Figure 4(a)). On the other hand, renal nephrin expression significantly decreased in the diabetic control group compared to the normal control group. In the taurine-treated diabetic group, nephrin levels increased significantly compared to both the diabetic and normal control groups (Figure 4(b)). The type IV collagen mRNA increased in diabetic control group (1.6 ± 0.8) compared to normal control (1 ± 0.5) and the increased levels were reduced in taurine-treated group (1.3 ± 0.4). The levels were not significant in each group (data not shown).

### 4.5. The Renal VEGF and Nephrin Expression in the Kidney

**Figure 5:**
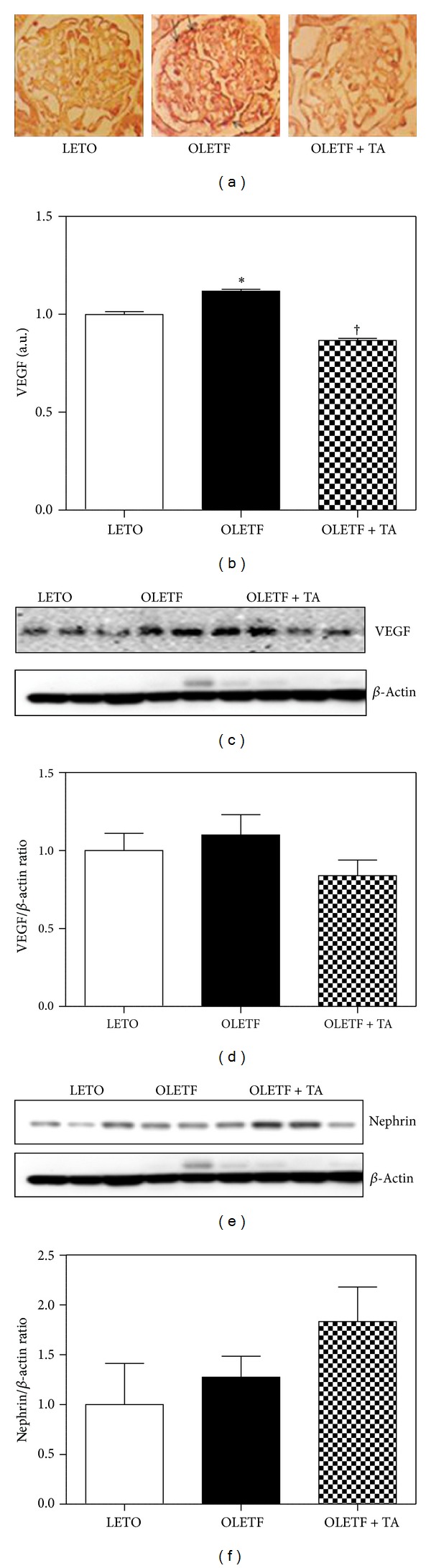
Effects of taurine on the expression of VEGF and nephrin in renal cortex. (a) Glomerular VEGF immunohistochemistry (arrows) in each group (×400). (b) Optical densities of VEGF in the glomeruli decreased significantly in the taurine-treated diabetic group compared to the diabetic control group. ((c), (d)) In the taurine-treated diabetic group, renal VEGF expression by western blot immunostaining decreased compared to that of the diabetic control group. ((e), (f)) The nephrin levels increased in taurine-treated group compared to diabetic control group. However the nephrin levels also increased in diabetic control group compared to normal control. But the VEGF and nephrin levels are not statistically significant. LETO, normal control group; OLETF, diabetic control group; OLETF + TA, taurine-treated diabetic group; VEGF, vascular endothelial growth factor. Data are expressed as mean ± SEM. *P < 0.05 compared with LETO; ^†^ *P* < 0.05 compared with OLETF.

By the immunohistochemistry, the renal VEGF expression decreased in the taurine-treated diabetic group compared to diabetic control group (Figures 5(a) and 5(b)) and by western blot, the VEGF expression decreased in the taurine-treated diabetic groups, although this difference was not statistically significant (Figures 5(c) and 5(d)). The nephrin levels increased in tuarine-treated diabetic group compared to diabetic control group. But the nephrin levels of diabetic control group also increased compared to normal control group. The levels were not significant different between each group (Figures 5(e) and 5(f)).

### 4.6. Antioxidant Effect of Taurine

**Figure 6:**
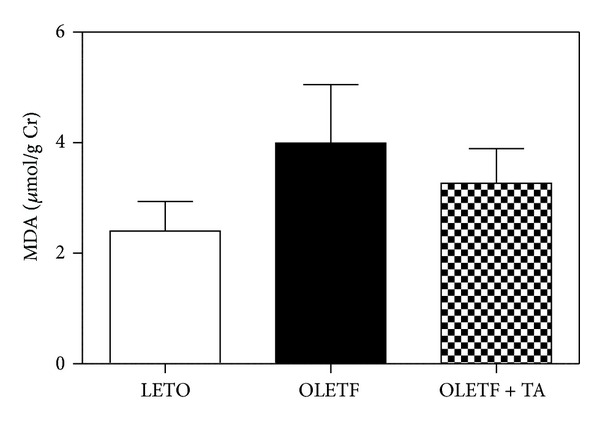
Changes in 24 hours urinary MDA levels at 45 weeks of age. In the diabetic control group, MDA increased compared to the normal control group. MDA decreased in the taurine-treatment group compared to the diabetic control group. However, there was no statistical significance in this difference. MDA, malondialdehyde; LETO, normal control group; OLETF, diabetic control group; OLETF + TA, taurine-treated diabetic group.

To determine the oxidative stress, we examined MDA levels in urine samples collected over 24 hours at 45 weeks of age. MDA in the diabetic control group increased compared to the normal control group. MDA was lower in the taurine-treated diabetic group than in the diabetic group, although this difference was not statistically significant (Figure 6).

### 4.7. The Effects of Taurine on VEGF, Nephrin, and ROS in Mouse Cultured Podocytes

**Figure 7:**
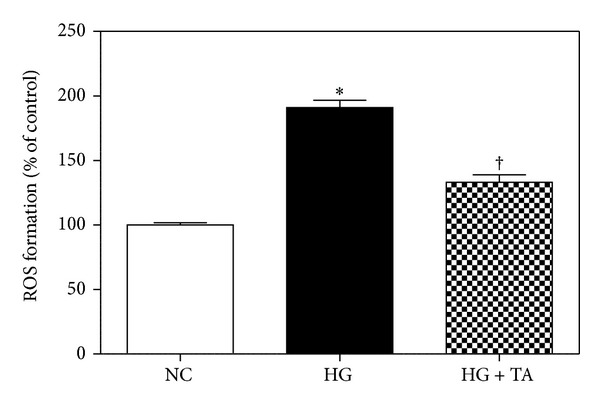
Changes in ROS formation in podocytes among the three groups. The taurine-treated high glucose group demonstrated a significantly decreased ROS production. NC, normal glucose; HG, high glucose; HG + TA, taurine-treated high glucose. Data are expressed as mean ± SEM. *P < 0.05 compared with NG; ^†^ *P* < 0.05 compared with HG.

**Figure 8:**
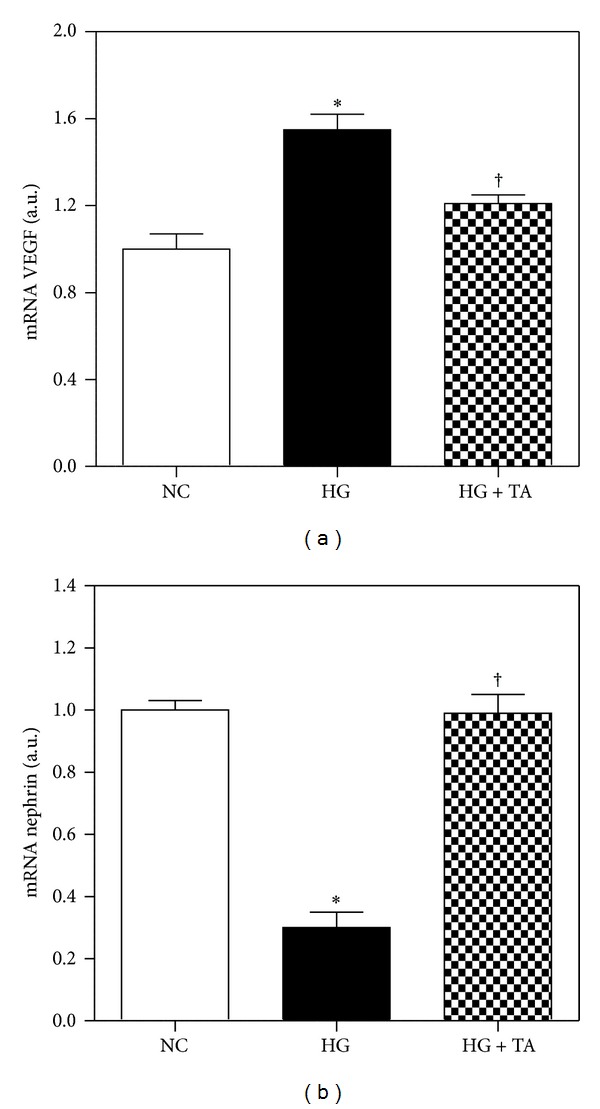
VEGF and nephrin mRNA expression by quantitative real-time PCR in mouse cultured podocytes. (a) VEGF mRNA expression significantly decreased in the taurine-treated HG group compared to that of the HG group. (b) In the taurine-treated high glucose group, nephrin expression increased compared to the high glucose group. NC, normal glucose; HG, high glucose; HG + TA, taurine-treated high glucose. Data are expressed as mean ± SEM. *P < 0.05 compared with NG; ^†^ *P* < 0.05 compared with HG.

ROS formation significantly decreased after taurine treatment (Figure 7). VEGF mRNA expression increased in podocytes treated with high glucose (HG) compared to those treated with normal glucose (NG) and nephrin mRNA expression reduced in mouse cultured podocytes treated with high glucose (HG) compared to those treated with normal glucose (NG). But the changes significantly recovered to the control level after taurine treatment (Figures 8(a) and 8(b)).

## 5. Discussion

Since Huxtable compiled the physiological actions of taurine in 1992, many studies of taurine have been reported [24]. Taurine is one of the most abundant amino acids in the mammalian organs, and has a potent antioxidant property [16, 26].

In diabetic nephropathy, taurine treatment reduces proteinuria and albuminuria and prevents glomerular hypertrophy [16, 17, 19], mesangial extracellular matrix expansion [20], and hypertrophy in renal tubular epithelial cells [27].

Our focus of this study was to understand molecular targets of taurine in diabetic nephropathy. Our data demonstrate that treating the diabetic group with taurine led to decreased ACR and also ameliorated the glomerular volume, GBM thickness, and the numbers of open slit pores compared to the diabetic control group. Increased oxidative stress is one of the reasons for the pathogenesis of diabetic nephropathy [28–30]. Several studies have also reported the prevention of diabetic renal disease after taurine treatment and its association with decreased ROS formation [16–20]. Our study showed that urinary MDA level was lower in the taurine-treated diabetic group than diabetic control group. However, there was no significant difference between the groups.

The diabetes control and complications trial (DCCT) and some clinical studies have demonstrated that lowering high blood glucose levels prevents the development and progression of diabetic renal disease [31, 32]. In our study, fasting blood glucose levels decreased significantly in the taurine-treated diabetic group compared to the diabetic control group, along with the increment of HOMA-*β* in the taurine treatment. Taurine may act as a regulator of insulin secretion [33], and hence the protective effect of taurine on diabetic nephropathy may be accomplished by blood glucose lowering through improved insulin secretion. Moreover, studies suggested that taurine treatment diminishes the rate of renal gluconeogenesis and also promoting the transformation of glucose to glycogen [18, 21, 34].

Nephrin is a podocyte-specific protein [12] and its reduction is related to increased glomerular hyperpermeability in diabetic nephropathy [35]. Diminished nephrin expression and altered nephrin localization were shown in patients with nephropathy in both type 1 and type 2 diabetes [13]. Nephrin gene expression varies according to glomerular size [36]. Changes in nephrin expression are associated with the extent of proteinuria in diabetic nephropathy [37]. Our data indicate that nephrin mRNA expression was increased significantly in the taurine-treated high glucose group compared to the high glucose group. This result implies that taurine prevent glomerular hyperpermeability through increased expression of nephrin. The other antioxidant agent, resveratrol (RSV) which is potent free radical scavenger, attenuates renal dysfunction and oxidative stress in STZ induced diabetic model rats and increases the nephrin levels in diabetic kidneys [38, 39].

VEGF is a major controller of angiogenesis and vascular permeability [40]. Renal VEGF is especially increased during the early stages of diabetic renal disease and reducing VEGF overexpression may ameliorate diabetic renal disease [8, 9]. Although the mechanism of VEGF-induced proteinuria in diabetic nephropathy is unclear, Unemori et al. observed the high vascular permeability by renal VEGF stimulates collagenase production and proteolytic disruption of the endothelial basement membrane [41]. Due to increased ROS in diabetic nephropathy, the VEGF and nephrin levels had been shown to be reduced or increased, respectively, to development or progression of proteinuria in diabetic nephropathy [42]. Sun et al. have shown that renal hypoxia and VEGF mRNA level consequently improve renal tubulointerstitial hypoxia of the diabetic rat kidney [43]. Nephrin expression is closely related to VEGF expression because VEGF signaling is essential for the formation and maintenance of a functional glomerular filtration barrier [44, 45].

In this study, not only ROS formation decreased significantly in the high glucose with taurine-treated group compared to the diabetic control group in podocytes but also decreased renal VEGF mRNA in both the kidney and podocytes.

We could not measure blood pressure in animal. In deed lowering arterial pressure is related to improvements in proteinuria. Harada et al. suggested taurine supplementation reduces hypertension in rat. Lowering arterial pressure is related to improvements in proteinuria [46].

## 6. Conclusion

Taurine may prevent the progression of diabetic nephropathy, possibly by its antioxidant property and also through the recovery in nephrin and reduction in renal VEGF expression.

## Figures and Tables

**Table 1:** Biochemical characteristics in experimental groups.

	LETO	OLETF	OLETF + TA
FBG (mg/dL)	90.1 ± 1.36	171.1 ± 14.32*	119.3 ± 10.94^†^
Insulin (ng/mL)	2.5 ± 0.65	1.0 ± 0.45*	1.6 ± 0.59^∗,†^
Kitt (%/min)	5.9 ± 0.36	1.6 ± 0.49*	2.3 ± 0.54*
HOMA-IR	30.38 ± 2.64	26.12 ± 3.30	27.9 ± 5.47
HOMA-*β*	1908 ± 249.4	258.7 ± 65.23*	726.8 ± 133.03^∗,†^
ADP (*μ*g/mL)	7.3 ± 1.12	5.9 ± 1.39*	6.6 ± 1.16

Data are expressed as mean ± SEM.; ADP: adiponectin; FBG: fasting blood glucose; HOMA-*β*: homeostasis model assessment for beta-cell function; HOMA-IR: homeostasis model assessment for insulin resistance; Kitt: short insulin tolerance test; LETO: normal control group; OLETF: diabetic control group; OLETF + TA: taurine-treated diabetic group. *P < 0.05 compared with LETO; ^†^ *P* < 0.05 compared with OLETF.
